# Resveratrol inhibits parathyroid hormone-induced apoptosis in human aortic smooth muscle cells by upregulating sirtuin 1

**DOI:** 10.1080/0886022X.2019.1605296

**Published:** 2019-05-20

**Authors:** Yingjie Liu, Yiru Wu, Zongli Diao, Weikang Guo, Wenhu Liu

**Affiliations:** Department of Nephrology, Faculty of Kidney Diseases, Beijing Friendship Hospital, Capital Medical University, Beijing, PR China

**Keywords:** Parathyroid hormone, sirtuin 1, resveratrol, apoptosis, smooth muscle

## Abstract

**Background:** Cardiovascular disease is the leading cause of death in patients with chronic kidney disease, so there is an urgent need to identify therapeutic targets to control the progression of cardiovascular disease. Apoptosis of aortic smooth muscle cells can promote cardiovascular disease, but the role of parathyroid hormone (PTH) and sirtuin 1 in the pathophysiology of apoptosis is still unclear.

**Methods:** Cultured human aortic smooth muscle cells (HASMCs) were stimulated with 10^–6^, 10^–8^, or 10^–10^ mol/L PTH for different days, apoptosis was measured by flow cytometry and sirtuin 1 and Bcl-2 protein levels in cell extracts were analyzed by western blotting. HASMCs were stimulated with PTH (10^–8^ mol/L) and 50 or 100 μmol/L RES for 3 d, apoptosis was measured by flow cytometry and sirtuin 1 and Bcl-2 protein levels in cell extracts were analyzed by western blotting.

**Results:** We found that PTH decreased the expression of sirtuin 1 and Bcl-2, inducing apoptosis (*p*<.05). Resveratrol (RES), a sirtuin 1 agonist, inhibited PTH-induced apoptosis and restored Bcl-2 expression (*p*<.05).

**Conclusions:** PTH induces apoptosis in HASMCs. Resveratrol inhibits PTH-induced apoptosis in HASMCs.

## Introduction

The risk of death from cardiovascular disease is increased in chronic kidney disease (CKD), mainly because of vascular calcification, which can develop by one of three mechanisms: cell transdifferentiation, cytotoxicity, and apoptosis. The formation of apoptotic bodies results in deposition of calcium and phosphorus, so understanding apoptosis may assist in preventing and treating cardiovascular events in CKD.

Secondary hyperparathyroidism from over-secretion of parathyroid hormone (PTH) is a common complication in patients with CKD, but there are few studies on PTH and cardiovascular calcification or apoptosis. PTH is the main regulator of blood calcium and phosphorus balance, and its functional impairment contributes to the development of cardiovascular disease-related pathologies.

Sirtuins, a family of nicotinamide adenine dinucleotide-dependent enzymes, are widely expressed in mammals and contain a highly conserved deacetylase domain. There are seven recognized sirtuins in mammals [1]. Due to their differential distribution in cells, they serve multiple functions in organisms. Silent mating type information regulation 2 homolog 1 (sirtuin 1) is homologous to SIR2, a silencing information regulator in yeast cells, and regulates some metabolic and aging processes in organisms. Previous studies have shown that sirtuin-1 can protect organs, such as the heart, liver, muscle, and adipose tissue, and regulates the metabolism of multiple target proteins [2]. Sirtuin 1 can prevent apoptosis by deacetylating nuclear transcription factors; such as p53, Ku70, and FOXO. Sirtuin 1 has also been shown to negatively regulate oxidative stress and cardiac remodeling [1,3–7]. Therefore, we hypothesized that up-regulation of sirtuin 1 expression in human aortic smooth muscle cells (HASMCs) can also protect organs.

Resveratrol (3,4,5-trihydroxystilbene) (RES) is a natural plant polyphenol and a specific agonist of sirtuin 1. Whether it can affect PTH-induced apoptosis in HASMCs is unknown. We hypothesized that sirtuin 1 may be a downstream target of PTH. The objective of the study was to assess whether PTH can induce apoptosis and whether sirtuin 1 is involved in PTH-induced apoptosis in HASMCs.

## Materials and methods

### Chemical reagents

PTH, RES, and sirtuin 1 were obtained from Sigma-Aldrich (St. Louis, MO). The apoptosis regulator Bcl-2 was purchased from Santa Cruz Biotechnology Inc. (Dallas, TX). All other reagents were purchased from Sigma-Aldrich (St. Louis, MO), unless specified otherwise.

### Cell culture

Primary HASMCs were obtained from ScienCell Research Laboratories Inc. (Carlsbad, CA). HASMCs were cultured in fibronectin‐coated flasks using smooth muscle cell medium (ScienCell, Carlsbad, CA) with BulletKit additives (ScienCell, Carlsbad, CA) and 10% (v/v) fetal bovine serum (ScienCell, Carlsbad, CA). HASMCs used in the experiments were at passages 3–4.

### Western blot analysis

Protein concentrations were determined using a BCA protein assay kit (Thermo Fisher Scientific, Waltham, MA). Total protein was extracted with lysis buffer (50 mM Tris–HCl, pH 7.4, 150 mM NaCl, 1% Triton X-100, 1% sodium deoxycholate, 0.1% SDS, and 1% phenylmethylsulfonyl fluoride). Samples were mixed with an equal volume of loading buffer (125 mM Tris–HCl, 4% SDS, 100 mM DTT, 20% glycerol, and 0.2% bromophenol blue) and heated at 99 °C for 10 min. Next, 20μg protein aliquots were resolved on 8–12% SDS-PAGE gel and transferred to nitrocellulose membranes by for electrophoresis (Amersham, GE Healthcare, Little Chalfont, UK). Membranes were blocked with 5% nonfat milk in Tris-buffered saline containing 0.1% Tween 20 for 2–3 h at room temperature, followed by an overnight incubation with primary antibodies at 4 °C. The membranes were then incubated with horseradish peroxidase-conjugated secondary antibodies for 1 h at room temperature, and exposed to an enhanced chemiluminescence kit (Millipore, Bedford, MA) and Kodak X-OMAT film (Eastman Kodak Inc., Rochester, NY). The primary antibodies used were anti-Bcl-2 (1:1000), anti-sirtuin 1 (1:10,000), and anti-GAPDH (1:1000).

### Apoptosis measurements

We incubated HASMCs in 6-well cell culture plates at a density of 1 × 10^5^ cells/mL at 37 °C for 2 d, until the bottoms of the plates were covered. The HASMCs were then counted, centrifuged at 1200×*g* for 5 min at room temperature, and cultured with 195 μL Annexin V-FITC binding solution and 5 μL Annexin V-FITC for 10 min at room temperature. Next, cells were assayed using an Annexin V-FITC apoptosis detection kit (Beyotime Institute of Biotechnology, Haimen, China).

### Statistical analysis

Data are expressed as means ± SE. Comparisons between experimental groups were made using one-way ANOVA. Differences in mean values were considered significant at *p*< .05.

## Results

### PTH treatment induces apoptosis

**Figure 1.**
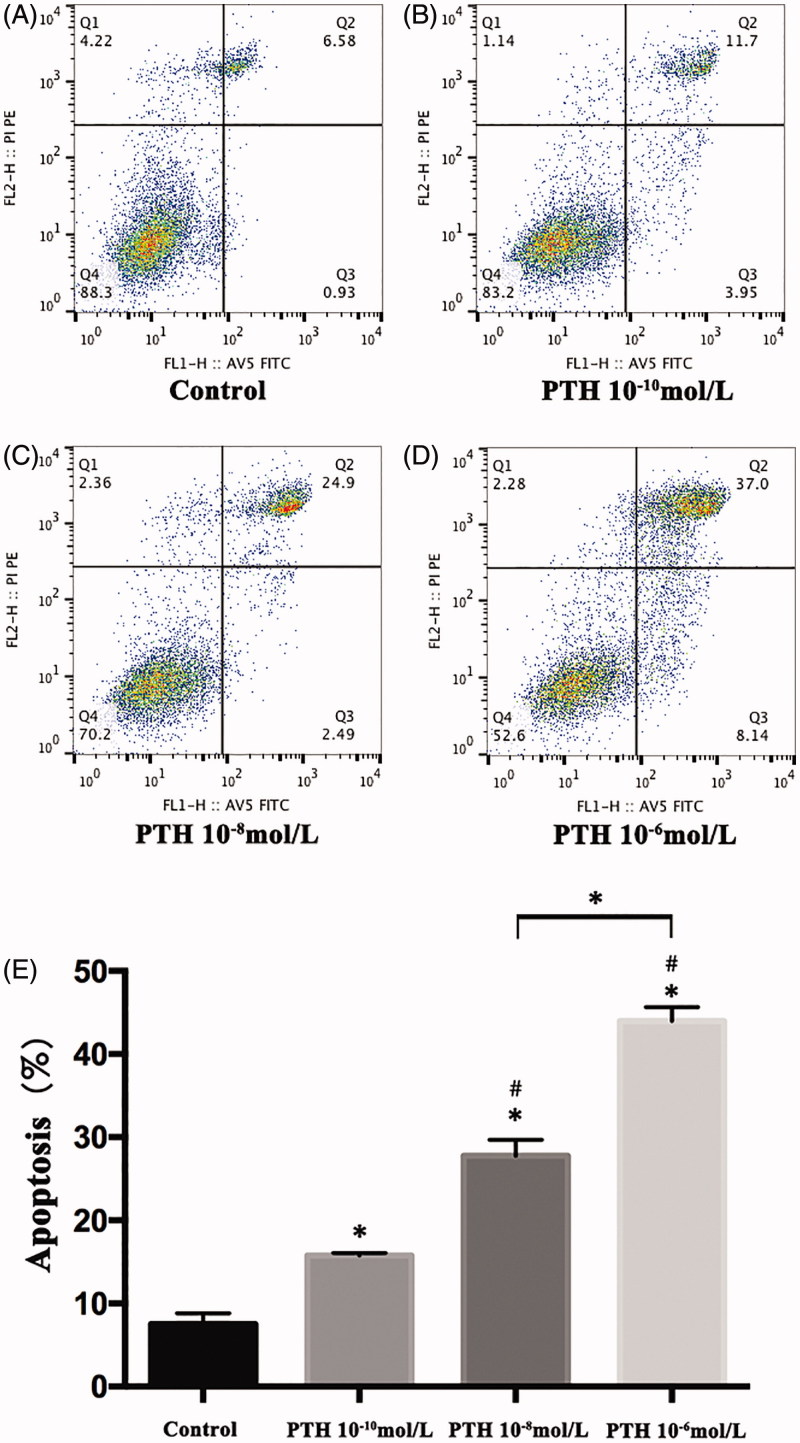
Parathyroid hormone (PTH) induces apoptosis in human aortic smooth muscle cells (HASMCs). HASMCs were stimulated with 10^−6^, 10^−8^, or 10^−10^ mol/L PTH for 3 d. Apoptosis was measured by flow cytometry. (A–D) Representative results of four experiments. (E) Results are presented as the mean ± SE of four independent experiments. *p< .05 compared with control. #*p*< .05 compared with the 10^−10^-mol/L PTH group.

**Figure 2.**
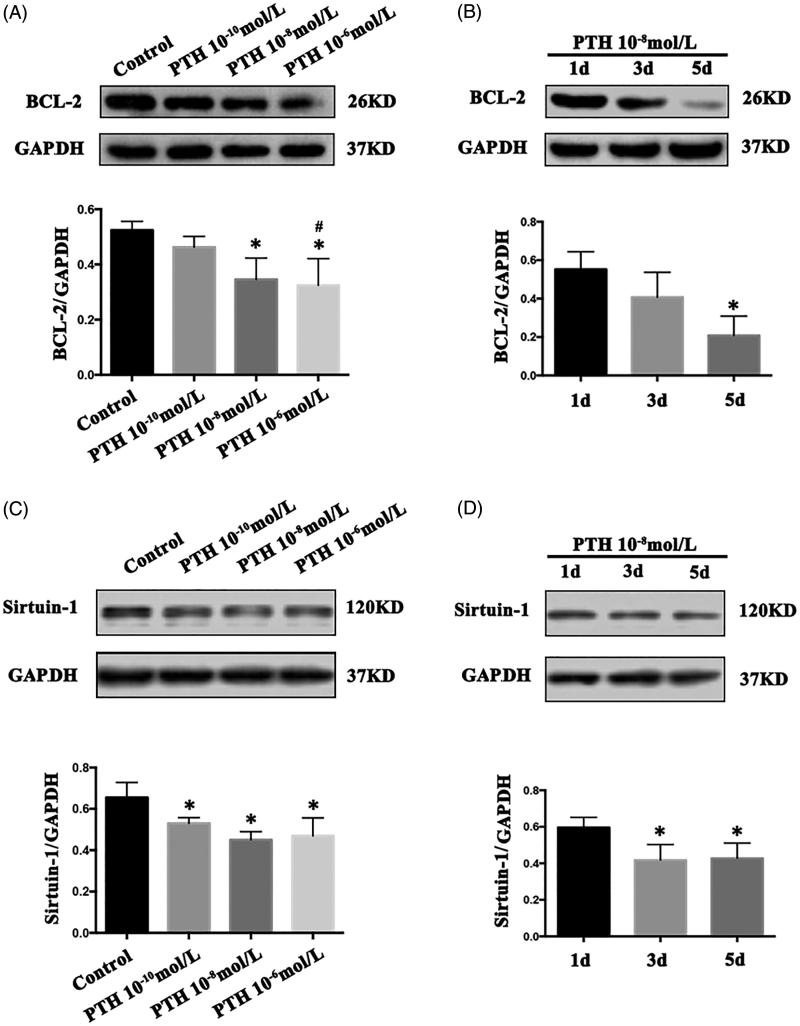
Parathyroid hormone (PTH) suppresses Bcl-2 and increases sirtuin 1 expression in human aortic smooth muscle cells (HASMCs). (A and B) Cells were treated with 10^−6^, 10^−8^, or 10^−10^ mol/L PTH for 3 d, or incubated with 10^−8^ mol/L PTH for 1, 3, or 5 d. Bcl-2 protein levels in cell extracts were analyzed by western blotting. (C and D) Cells were treated with PTH and sirtuin 1 levels were measured. Results are presented as the mean ± SE (*n* = 3). *p< .05 compared with control. #*p*< .05 compared with the 10^−10^-mol/L PTH group.

The proportion of apoptotic cells was measured by flow cytometry. HASMCs treated with PTH (10^−6^, 10^−8^, or 10^−10^ mol/L) had a significantly higher proportion of apoptotic cells than control cells after 3 d, and apoptosis was concentration-dependent (*p*< .05, Figure 1). The expression of Bcl-2, an apoptosis inhibitor, was downregulated significantly: when HASMCs were incubated with 10^−6^ or 10^−8^ mol/L PTH for 3 d, Bcl-2 levels decreased compared with levels in the controls (*p*< .05, Figure 2(A)), but no such effect was observed in cells exposed to 10^−10^ mol/L PTH. We therefore concluded that 10^−8^ mol/L PTH is the most suitable low concentration for inducing apoptosis in HASMCs. In cells incubated with 10^−8^ mol/L PTH for 1, 3, and 5 d, Bcl-2 was significantly downregulated in a time-dependent manner (*p*< .05, Figure 2(B)).

### Sirtuin 1 is involved in PTH-induced apoptosis in HASMCs

We tested the involvement of sirtuin 1 in PTH-induced apoptosis in HASMCs. Levels of sirtuin 1 decreased following incubation of HASMCs with 10^−6^, 10^−8^, or 10^−10^ mol/L PTH for 3 d (*p*< .05, Figure 2(C)). In HASMCs incubated with 10^−8^ mol/L PTH for 1, 3, or 5 d, sirtuin 1 levels decreased in a time-dependent manner (*p*< .05, Figure 2(D)). As expected, PTH downregulated the expression of sirtuin 1 in HASMCs.

### Resveratrol inhibits PTH-induced apoptosis in HASMCs

**Figure 3.**
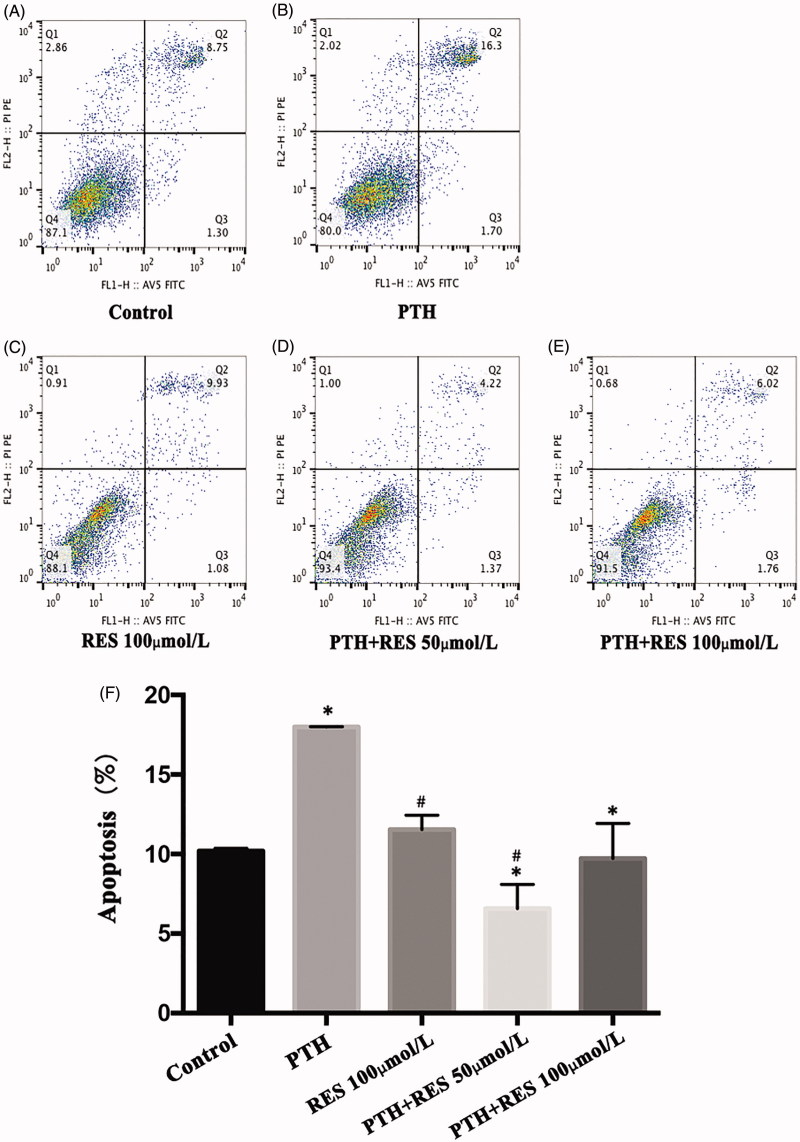
Resveratrol (RES) inhibits parathyroid hormone (PTH)-induced apoptosis in human aortic smooth muscle cells (HASMCs). HASMCs were stimulated with PTH (10^−8^ mol/L) and 50 or 100 μmol/L RES for 3 d. Apoptosis was measured by flow cytometry. (A–D) Representative results of four experiments. (E) Results are presented as the mean ± SE of four independent experiments. *p< .05 compared with control. #*p*< .05 compared with the PTH group.

**Figure 4.**
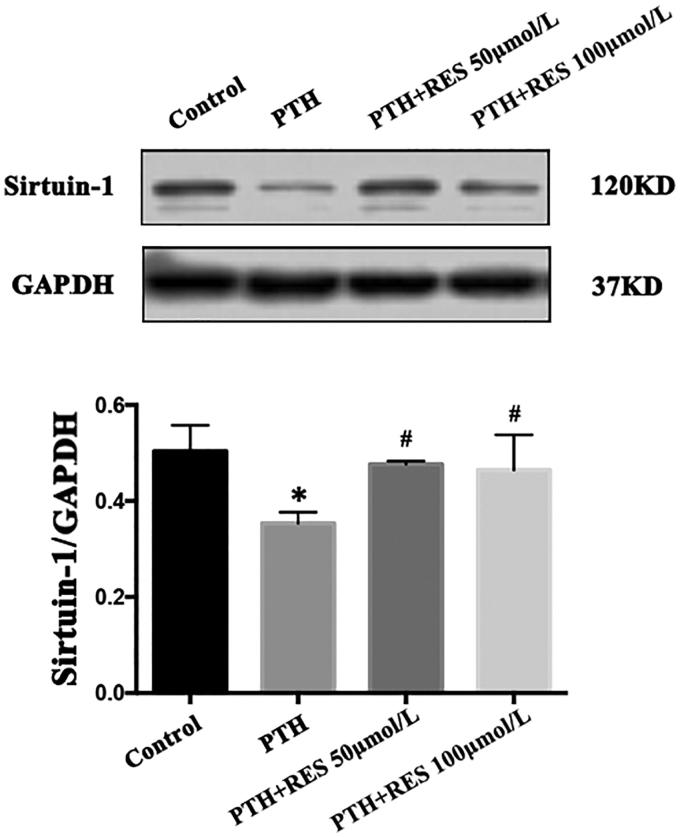
Resveratrol (RES) increases sirtuin 1 expression in human aortic smooth muscle cells (HASMCs). Cells were treated with parathyroid hormone (PTH) and 50 or 100 μmol/L RES for 3 d. Sirtuin 1 protein levels in cell extracts were analyzed by western blotting. Results are presented as the mean ± SE (*n* = 3). *p< .05 compared with control. #*p*< .05 compared with the PTH group.

Resveratrol is a specific agonist of sirtuin 1, so we assessed whether RES can decrease the PTH-induced apoptosis in HASMCs. When we co-cultured cells with RES (50 or 100 μmol/L) and 10^−8^ mol/L PTH for 3 d, we found that RES increased the levels of sirtuin 1, but not in a concentration-dependent manner (Figure 3). Compared with apoptosis in the control group (no RES), the RES-treated cells exhibited decreased apoptosis (*p*< .05), but not in a concentration-dependent manner (Figure 4). These results indicate that RES can inhibit PTH-induced apoptosis in HASMCs by upregulating sirtuin 1.

## Discussion

Cardiovascular disease is the leading cause of death in CKD patients. There is an urgent need to identify therapeutic targets to control the progression of cardiovascular disease. We wanted to investigate the relationship between PTH and vascular calcification, so we used PTH to stimulate human aortic smooth-muscle cells and alizarin red staining and silver nitrate staining to detect interstitial calcium deposition. However, there were no positive results in many of the experiments. We suspect that PTH did not affect vascular calcification to a notable extent. We then used flow cytometry and western blotting to detect apoptosis, hoping to find a link between PTH and apoptosis.

Apoptosis is an important mechanism of vascular calcification; we investigated the relationship between PTH, apoptosis, and sirtuin 1, and found that PTH can promote apoptosis in a concentration-dependent manner, while co-culturing with RES inhibited apoptosis.

PTH is an important regulator of calcium and phosphate homeostasis, and affects the function of cells from mineralized tissues such as osteoblasts [8], osteocytes [9], and odontoblasts. PTH is a polypeptide that acts on the skeletal system and renal tubules to regulate serum calcium and phosphorus. PTH can promote the proliferation and differentiation of osteoblasts and reduce the apoptotic rate of osteoblasts [10]. Under normal conditions, bone resorption is equal to bone formation [11], and PTH can alter this balance, resulting in increased bone turnover. Some PTH formulations have been shown to reduce the risk of vertebral fractures, and are used in treating osteoporosis in postmenopausal women [12]. We used super-physiological concentrations of PTH to stimulate apoptosis, and found concentration-dependent increases in programed cell death. Excessive PTH may therefore cause vascular calcification through apoptosis, and this effect requires further study.

The specific mechanism is still unknown. PTH may bind to the PTH receptor and act on intracellular components to induce apoptosis. Activation of PTH or the PTH-related protein of the PTH1R gene results in a sharp increase in several intracellular signaling molecules, including the enzymes adenylate cyclase and phospholipase C [13,14]. Although the role of PTH in osteocytes has been frequently evaluated, few studies have been carried out in HASMCs.

An increase in osteoblasts has been shown to be driven by a reduction in apoptosis; this reduction is regulated by cAMP/PKa and can lead to the transcription of the anti-apoptotic Bcl-1 and 2 and the inactivation of pro-apoptotic genes [10].

We found that PTH induces apoptosis by downregulating sirtuin 1, a member of the sirtuin deacetylase family that regulates cell senescence and energy metabolism.

Resveratrol is a naturally occurring diphenol; there is a large body of evidence for its beneficial anticancer, antioxidant, anti-inflammatory, and cardioprotective effects in organisms [15,16]. Resveratrol has been shown to prevent the development of cardiac hypertrophy and dysfunction in spontaneously hypertensive rats [17]. We used RES as a sirtuin 1 agonist, and found that it inhibited PTH-induced apoptosis in HASMCs. We plan to continue this line of investigation in animal experiments.

## Conclusions

PTH-induced apoptosis is associated with decreases in sirtuin 1 expression, and RES can prevent this decrease. Therapies that target impaired endogenous sirtuin 1 or involve RES supplementation may constitute a novel approach for treating PTH-induced vascular injury.

## Data Availability

The analyzed data sets generated during the study are avail- able from the corresponding author on reasonable request.
